# Development of human monoclonal antibodies against TARM1 by yeast display

**DOI:** 10.1002/2211-5463.70216

**Published:** 2026-04-09

**Authors:** Rikio Yabe, Mayumi Saeki, Masaaki Hashiguchi, Sayaka Ono, Kazuhisa Aoki, Hidetaka Tanno

**Affiliations:** ^1^ Cancer Immunology Project Tokyo Metropolitan Institute of Medical Science Setagaya Japan; ^2^ Graduate School of Medical and Dental Sciences Institute of Science Tokyo Japan

**Keywords:** fully human monoclonal antibody, TARM1, yeast display

## Abstract

TARM1, a leukocyte immunoglobulin‐like receptor expressed on myeloid cells, functions as a costimulatory receptor promoting proinflammatory cytokine secretion. Recent studies have revealed the involvement of TARM1 in immune‐mediated diseases. Despite its biological importance, research tools for elucidating human TARM1 function have been limited. Here, we generated monoclonal antibodies (mAbs) against human TARM1 by a yeast display platform combined with a human single‐chain variable fragment (scFv) library. Two scFv clones were isolated and converted into IgG1 antibodies, both of which bound recombinant TARM1 with high affinity and cell‐surface TARM1. Importantly, these antibodies induced activation signals into Jurkat NFAT‐GFP reporter cells expressing TARM1–CD28–4‐1BB–CD3ζ chimera, indicating agonistic activity. These mAbs provide valuable tools for dissecting TARM1‐mediated function and represent a potential approach for therapeutic strategies in inflammatory diseases and cancer.

## Graphical abstract

Human monoclonal antibodies against TARM1 are generated by yeast display‐guided selection. These antibodies bind to soluble and cell‐surface forms of TARM1. Also, these antibodies exhibit agonistic activity in the NFAT‐GFP reporter assay, indicating that TARM1 signaling can be functionally modulated by antibodies and suggesting TARM1 as a potential immunomodulatory target for immunotherapy.

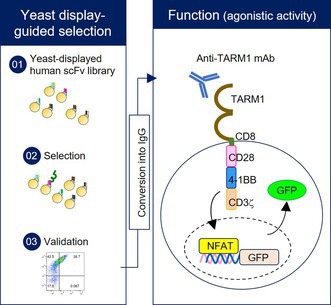

**Abbreviations**
ABCD: antibodies chemically defined
AF: Alexa Fluor
BFP: monomeric blue fluorescent protein with improved brightness and chemical stability
BLI: biolayer interferometry
BV: Brilliant Violet
CITA: clone isolated by TARM1
FITC: fluorescein isothiocyanate
GFP: green fluorescent protein
GH1: growth hormone 1
Ig: immunoglobulin
ITAM: immunoreceptor tyrosine‐based activation motif
ITIM: immunoreceptor tyrosine‐based inhibitory motif
LILR: leukocyte immunoglobulin‐like receptor
LRC: leukocyte receptor complex
mAb: monoclonal antibody
MACS: magnetic‐activated cell sorting
MHC: major histocompatibility complex
MTA: material transfer agreement
NFAT: nuclear factor of activated T cells
PBMC: peripheral blood mononuclear cell
PBS: phosphate‐buffered saline
scFv: single‐chain variable fragment
TARM1: T‐cell‐interacting, activating receptor on myeloid cells protein 1
VH: variable heavy chain
VL: variable light chain
WCL: whole cell lysate

The leukocyte immunoglobulin‐like receptor (LILR) family consists of a large set of activating and inhibitory receptors expressed predominantly on myeloid and lymphoid lineage cells [1, 2, 3]. These receptors share structural features, including extracellular immunoglobulin (Ig)‐like domains and transmembrane regions. Additionally, inhibitory‐type LILRs typically contain immunoreceptor tyrosine‐based inhibitory motifs (ITIMs) in their cytoplasmic tails, whereas activating‐type LILRs often signal through association with the immunoreceptor tyrosine‐based activation motif (ITAM)‐bearing Fc receptor γ‐chain [4]. LILRs regulate both innate and adaptive immune responses upon recognition of ligands such as major histocompatibility complex (MHC) class I molecules or pathogen‐derived structures [5]. The balance between activating and inhibitory signaling is essential for maintaining immune homeostasis, while dysregulation of this system contributes to autoimmunity, chronic inflammation, and impaired host defense [6].

T cell‐interacting, activating receptor on myeloid cells‐1 (TARM1) is a LILR family member encoded within the leukocyte receptor complex (LRC)– a genomic region on chromosome 19q13.4 that contains multiple immunoglobulin‐like receptor genes involved in immune regulation [7]. TARM1 consists of two extracellular Ig‐like domains, a transmembrane domain, and a short cytoplasmic tail. It is predominantly expressed on myeloid lineage cells, including neutrophils and monocytes [8]. TARM1 associates with an adaptor protein Fc receptor γ‐chain bearing ITAM and transduces activation signals. TARM1 functions as a costimulatory receptor that promotes proinflammatory cytokine secretion in response to innate immune stimuli.

TARM1 plays an important role in the development of various immune‐mediated diseases. We recently reported that TARM1 is involved in the development of arthritis by enhancing dendritic cell activation and promoting T‐cell responses [9]. TARM1 also contributes to host responses against *Mycobacterium tuberculosis* infection [10, 11]. In addition, TARM1 is suggested to affect colitis by regulating macrophage M1 polarization in a mouse colitis model [12]. More recently, TARM1 was implicated in acute kidney injury, where macrophage autophagy protected against renal inflammation through the degradation of TARM1 [13]. These findings suggest that TARM1 is a promising therapeutic target for modulating immune responses.

Despite its biological importance and therapeutic relevance, research tools for elucidating human TARM1 function have been limited. A previous study reported the generation of a monoclonal antibody (mAb) against murine TARM1 [8]. However, no human‐type antibodies have yet been reported. Given the translational potential of targeting TARM1 in immune‐mediated diseases, the development of fully human or humanized TARM1‐specific antibodies is important for both mechanistic studies and future clinical applications.

In the present study, we report the generation and characterization of novel human TARM1‐specific mAbs using a human single‐chain variable fragment (scFv)‐expressing yeast display platform. We reveal that two mAbs specifically bind to human TARM1, recognize TARM1 expressed on human cells, and possess agonistic activity.

## Materials and methods

### Ethics statement

All experiments involving recombinant DNA were approved by the Recombinant DNA Experiments Committee of the Tokyo Metropolitan Institute of Medical Science (approval ID. 23‐039). All procedures involving human‐derived materials were approved by the Research Ethics Committee of the same institute (approval ID. 21‐43). The study was performed in accordance with the Declaration of Helsinki. The human scFv library used in this study was constructed from peripheral blood mononuclear cells (PBMCs) of healthy donors, which were previously purchased from Lonza (#CC‐2704). Lonza confirmed that these PBMCs were collected from donors who had provided written informed consent for research use.

### Plasmids

Human TARM1 cDNA was obtained from R&D Systems (#RDC2334; Minneapolis, MN, USA). For construction of pHEK‐TARM1‐Fc, a synthetic gene coding a growth hormone 1 (GH1) signal peptide, the TARM1 ectodomain and human IgG1 Fc region was cloned into pHEK293 Ultra Expression Vector I (#3390; Takara, Tokyo, Japan) using NEBuilder HiFi DNA Assembly Master Mix (#E2621X; New England Biolabs, Ipswich, MA, USA). For construction of an antibody expression vector, a GH1 signal peptide, variable heavy (VH) and light (VL) chain sequences obtained from round‐4 sorted yeast clones, and the IgHG1 or IgL2 constant regions were assembled into pHEK293 Ultra Expression Vector I. A gene encoding a CD8 signal peptide, Flag tag, TARM1 ectodomain and transmembrane region, T2A sequence and emerald green fluorescent protein (emGFP) was cloned into a pMYs‐IRES‐GFP (pMYs‐IG)–derived backbone lacking the IRES‐GFP cassette (kind gift from Toshio Kitamura, University of Tokyo). A fragment coding a CD8 signal peptide, Flag tag, TARM1 ectodomain, CD8 hinge and transmembrane regions, CD28–4‐1BB–CD3ζ signaling domains and T2A‐monomeric blue fluorescent protein with improved brightness and chemical stability (BFP) was synthesized (Twist Bioscience, South San Francisco, CA, USA). The fragment was cloned into a pMYs‐IG–derived backbone lacking the IRES‐GFP cassette. The resulting construct is hereafter referred to as Flag‐TARM1‐chimera. pSIRV‐nuclear factor of activated T cells (NFAT)‐GFP (Addgene #118031, gift from Peter Steinberger), pMD2.G (Addgene #12259, gift from Didier Trono), and pCTcon2 (Addgene #41843, gift from Dane Wittrup) were obtained from Addgene. All constructs were sequence‐confirmed.

### Cell culture

293FT (RRID:CVCL_6911) and Expi293F cells (RRID:CVCL_D615) were purchased from Thermo Fisher Scientific (R70007 and A14635; Waltham, MA, USA). Plat‐GP cells (RRID:CVCL_B490) are a kind gift from Toshio Kitamura, University of Tokyo. Jurkat cells (RRID:CVCL_0065) were purchased from Riken Cell Bank (#RCB3052; Tsukuba, Japan). 293FT, Plat‐GP, and Flag‐TARM1‐293FT cells were cultured with DMEM supplemented with 10% inactivated FBS and 100 U·mL^−1^ penicillin and 100 μg·mL^−1^ streptomycin. Expi293F cells were cultured with Expi293 Expression Medium (A1435101; Thermo Fisher Scientific). Jurkat NFAT‐GFP and Flag‐TARM1‐chimera‐Jurkat NFAT‐GFP cells were cultured with RPMI1640 supplemented with 10% inactivated FBS and 100 U·mL^−1^ penicillin and 100 μg·mL^−1^ streptomycin. All experiments were conducted using cells confirmed to be free of mycoplasma contamination. All cell lines used in this study were obtained from established commercial or public repositories and were used shortly after receipt. No independent cell line authentication was performed by the authors.

### Preparation of monocyte‐derived dendritic cells

CD14^+^ monocytes were purified from human PBMCs (#39000‐80M; Fuji film Wako, Osaka, Japan) by positive magnetic selection using CD14 MicroBeads (Miltenyi Biotec, Bergisch Gladbach, Germany) according to the manufacturer's instructions. Purified CD14^+^ cells (purity 95%) were seeded at 1.25 × 10^6^ cells·mL^−1^ in RPMI1640 supplemented with 10% FBS, 100 U·mL^−1^ penicillin, and 100 μg·mL^−1^ streptomycin, 100 ng·mL^−1^ recombinant human granulocyte‐macrophage colony‐stimulating factor (#300‐03; GM‐CSF, Peprotech, Rocky Hill, NJ, USA) and 50 ng·mL^−1^ recombinant human interleukin‐4 (#200‐04; IL‐4, Peprotech). On day 3, the same volume of fresh R10 medium containing 200 ng·mL^−1^ GM‐CSF and 100 ng·mL^−1^ IL‐4 was added to the dishes. On day 6, immature monocyte‐derived dendritic cells (moDCs) were collected and used for subsequent experiments.

### Retroviral transduction

Retroviruses were generated by co‐transfecting Plat‐GP cells with retroviral vectors (e.g., pMYs vector–based constructs) and pMD2.G using PEI MAX (#24765; Polyscience, Warrington, PA, USA). After 8 h of incubation, medium was changed. Virus‐containing supernatants were collected at 2‐ or 3‐day post‐transfection, filtrated using a 0.45 μm filter and frozen at −80 °C until use. Cells were infected with retrovirus supernatants in the presence of 8 μg·mL^−1^ polybrene. Twenty‐four hours later, medium was replaced with appropriate fresh media.

Jurkat NFAT‐GFP cells [14] were established by retroviral transduction of parental Jurkat cells with pSIRV‐NFAT‐GFP and pMD2.G, followed by single‐cell culture. To generate Flag‐TARM1‐chimera–Jurkat NFAT‐GFP cells, Jurkat NFAT‐GFP cells were transduced with retrovirus encoding the Flag‐TARM1‐chimera.

### Protein expression and purification

Plasmids were transfected into Expi293F cells by PEI MAX. After incubation for 16–18 h, the cell media were supplemented with 5 mm valproic acid, 6.5 mm sodium propionate and 0.9% glucose. Seven days later, culture supernatants were collected and filtrated through a 0.45 μm membrane filter. Purification of TARM1‐Fc, Fc, and mAbs was performed as previously described with some modification [15]. Culture supernatants were incubated with Ab‐Capcher‐beads (#P‐002‐10; ProteNova, Kagawa, Japan) at 4 °C overnight. After the beads were washed with phosphate‐buffered saline (PBS), fractions were eluted with Gly‐HCl (pH 2.8), and neutralized with 1 m Tris/HCl (pH 8.0). The purified proteins were desalted and concentrated using an Amicon Ultra‐15 (#UFC901024; 10 K MWCO, Merck, Darmstadt, Germany). The concentrations of proteins were measured using a spectrophotometer, DS‐11+ (DeNovix, Wilmington, DE, USA).

### Yeast display‐guided selection

A human scFv yeast‐display library previously prepared in our laboratory was used in this study [16]. Briefly, the library was generated from VH and VL repertoires amplified by error‐prone PCR using B cell‐derived cDNA from four healthy human donors as templates. The scFv amplicons were co‐electroporated with linearized pCTcon2 (6 μg; 7 kbp; 8 × 10^11^ molecules) into 1.6 × 10^9^ AWY101 yeast cells, as described previously [17]. Although colony counting was not performed at the time of construction or in the present study, the amount of DNA and the number of yeast cells used in this electroporation protocol are expected to yield approximately 10^8^–10^9^ independent clones.

Yeast display‐guided selection was performed as described previously [18]. Yeasts were cultured in SDCAA/Pc/Sm medium [20 g·L^−1^ Glucose (#049‐31165; Fuji film Wako)], 6.7 g·L^−1^ Difco yeast nitrogen base w/o amino acids [#291940; BD, Franklin Lakes, NJ, USA], 5 g·L^−1^ Acid hydrolysate of casein [#A1404HA; BIOKAR Diagnostics, Paris, France], 5.4 g·L^−1^ Na_2_HPO_4_ and 9.68 g·L^−1^ NaH_2_PO_4_*2H_2_O and Penicillin–Streptomycin [#168‐23191; Pc/Sm, Fujifilm Wako] at 30 °C overnight. The yeasts (OD600 of 100) were cultured in SGDCAA/Pc/Sm medium (2 g·L^−1^ Glucose, 6.7 g·L^−1^ Difco yeast nitrogen base w/o amino acids, 5 g·L^−1^ Acid hydrolysate of casein, 5.4 g·L^−1^ Na_2_HPO_4_, 9.68 g·L^−1^ NaH_2_PO_4_*2H_2_O, 20 g·L^−1^ galactose and Pc/Sm) at 20 °C for 2 days for the induction of cell‐surface scFvs. Multiple selection rounds of a library were performed as follows. For the first round of selection, yeasts (OD600 of 200) were incubated with 5 μg·mL^−1^ TARM1‐Fc at room temperature for 1 h in a platform shaker with gentle agitation. After washing with rinsing buffer [autoMACS Rinsing Solution (#130‐091‐222; Miltenyi Biotec) supplemented with MACS bovine serum albumin (BSA) Stock Solution (#130‐091‐376; Miltenyi Biotec)], yeasts were incubated with fluorescein isothiocyanate (FITC)‐anti‐human IgG Fc antibody (1 : 1000, M1310G05, #410720; BioLegend, San Diego, CA, USA) at room temperature for 30 min. After washing with rinsing buffer, the yeasts were incubated with Anti‐FITC MicroBeads (1 : 20, #130‐048‐701; Miltenyi Biotec) at 4 °C for 30 min. The yeast/bead mixture was washed with rinsing buffer and loaded onto the autoMACS Pro Separator system (Miltenyi Biotec). Sorted yeasts were washed with SDCAA/Pc/Sm medium, and were cultured in SDCAA/Pc/Sm medium at 30 °C for 2 days. For the second round of selection, negative selection was performed as described below. Yeasts (OD600 of 10) were cultured in SGDCAA/Pc/Sm medium for 2 days. Induced yeasts (OD600 of 100) were incubated with 5 μg·mL^−1^ Fc at room temperature for 1 h followed by FITC‐anti‐human IgG Fc antibody at room temperature for 30 min. Labeled yeasts were incubated with Anti‐FITC MicroBeads, and the yeast/bead mixture was subjected to AutoMACS sorting. An unsorted fraction was further sorted with 5 μg·mL^−1^ TARM1‐Fc followed by Alexa Fluor647 (AF647)‐anti‐human IgG Fc antibody (1 : 1000, M1310G05, #410714; BioLegend) and Anti‐Cy5/Anti‐AF647 MicroBeads (1 : 20, #130‐091‐395; Miltenyi Biotec). The third round of selection was performed by flow cytometry sorting. Induced yeasts (OD600 of 10) were subjected to negative selection as described in the second round, and unsorted yeasts were incubated with 5 μg·mL^−1^ TARM1‐Fc followed by Brilliant Violet 421 (BV421)‐anti‐human IgG Fc antibody (1 : 500, M1310G05, #410704; BioLegend) and AF647‐anti‐Myc antibody (PL14, 1 : 1000, #M047‐A64; MBL, Tokyo, Japan). TARM1‐Fc^+^Myc^+^ yeasts were sorted using a flow cytometer, SH800 (SONY, Tokyo, Japan). For the fourth round of selection, negative selection was performed as described in the second round, and an unsorted fraction was sorted by autoMACS with 1 μg·mL^−1^ TARM1‐Fc followed by AF647‐anti‐human IgG Fc antibody and Anti‐Cy5/Anti‐AF647 MicroBeads. After the fourth round, yeasts were spread onto SDCAA/Pc/Sm agar plates. Two days later, single colonies were cultured in SDCAA/Pc/Sm medium. The yeast expression plasmid pCTcon2 were isolated from the cells using Zymoprep Yeast Plasmid Miniprep Kit II (#D2004; Zymo Research, Irvine, CA, USA), and were further transformed into ElectroMAX Stbl3 Competent Cells (#11635018; Thermo Fisher Scientific). Single colonies were cultured in LB broth containing ampicillin. The plasmids were obtained from *Escherichia coli* using NucleoSpin Plasmid EasyPure (#U0727C; Takara) and Sanger‐sequenced.

### Flow cytometry

For binding analysis of scFv‐expressing yeast cells, yeasts (OD600 of 0.05) were incubated with 1 μg·mL^−1^ TARM1‐Fc or Fc on ice for 30 min. After washing with rinsing buffer, the yeasts were incubated with FITC‐anti‐human IgG Fc antibody (1 : 100, M1310G05, #410720; BioLegend) and AF647‐anti‐Myc‐tag antibody (1 : 100, PL14, #M047‐A64; MBL) on ice for 30 min. The yeasts were washed and analyzed using a flow cytometer, FACSCantoII (BD Biosciences, San Jose, CA, USA). For staining of TARM1 on cells, Flag‐TARM1‐293FT or 293FT cells were incubated with 1 μg·mL^−1^ anti‐TARM1 mAbs or human IgG1 (#403502, hIgG1; BioLegend) on ice for 30 min. After washing with rinsing buffer, the cells were incubated with 0.5 μg·mL^−1^ allophycocyanin (APC)‐anti‐human IgG Fc antibody (#410712, M1310G05; BioLegend) on ice for 30 min. Also, Flag‐TARM1‐293FT or 293FT cells were stained with 1 μg·mL^−1^ BV421‐anti‐DYKDDDDK antibody (#637321, L5; BioLegend). Cells were washed with rinsing buffer and analyzed by flow cytometry. Data analysis was performed using facsdiva version 6.1.2 (BD Biosciences) and flowjo Version 10 (BD Biosciences). Dead mammalian cells were stained with 1 μg·mL^−1^ 7‐amino‐actinomycin D (#420403, 7AAD; BioLegend). For isolation of Flag‐TARM1‐expressing 293FT cells, emGFP^+^ 293FT cells were sorted using flow cytometers, FACSAria III (Becton Dickinson, Franklin Lakes, NJ, USA).

### ELISA

Anti‐TARM1 mAbs (1 μg·mL^−1^) or hIgG1 were precoated on MaxiSorp 96‐well plates (#442404; Thermo Fisher Scientific) at 4 °C overnight. The plates were washed with PBS/0.05% Tween20 (PBS‐T) and blocked with 2% BSA/PBS at room temperature for 1 h. After washing, biotin‐TARM1‐Fc or biotin‐Fc (1, 3, and 10 μg·mL^−1^) were incubated at room temperature for 2 h. After washing, wells were incubated with Horseradish peroxidase (HRP)‐Streptavidin (#405210, 1 : 3000; BioLegend) at room temperature for 1 h. After washing, ELISA POD Substrate TMB solution (Easy) (#05299‐54; NACALAI TESQUE, Kyoto, Japan) was added and stopped with 1 N HCl (#18322‐95; NACALAI TESQUE). The absorbance at wavelength 450 nm was measured using a microplate reader, arvo x3 (PerkinElmer, Waltham, MA, USA).

### Western blot

Cells were incubated with lysis buffer [20 mm Tris/HCl buffer pH 7.4, 100 mm NaCl, 50 mm NaF, 0.5% NP‐40, 1 mm EDTA, and Protease Inhibitor Cocktail for Use with Mammalian cell and Tissue Extracts (#25955; Nacalai Tesque)] at 4 °C for 1 h. Then, cells were lysed by ultrasonication. After centrifugation at 20 000× ***g*** at 4 °C, the resulting supernatant was subjected to SDS/PAGE and electroblotting analyses onto a polyvinylidene difluoride (PVDF) membrane. The membrane was blocked with 5% BSA/PBS‐T (PBS containing 0.05% Tween 20) and incubated with 1 μg·mL^−1^ anti‐TARM1 mAbs CITA‐001, CITA‐006, or anti‐DYKDDDDK mAb (#014‐22383, 1E6; Fujifilm Wako) for 1 h at room temperature. After washing with PBS‐T, the membranes were incubated with HRP‐anti‐human IgG (H + L) (1 : 5000, #SA00001‐17; Proteintech, Rosemont, IL, USA) or HRP‐anti‐mouse IgG (H + L) (1 : 5000, Poly4053, #405306; BioLegend) for 1 h at room temperature. Whole cell lysates (WCLs) were also immunoblotted. The membranes were visualized with Western Lightning Pro, Chemiluminescent Substrate (#NEL120001EA; Revvity, Waltham, MA, USA). Chemiluminescent signal images were acquired using a digital imager, imagequant las500 system (Cytiva, Tokyo, Japan).

### Immunoprecipitation‐western blot

WCLs were precleared with Protein G‐Sepharose (# 11719416001; Roche, Basel, Switzerland). Then the supernatant was incubated with anti‐TARM1 mAbs CITA‐001, CITA‐006, or anti‐DYKDDDDK mAb (1 μg)‐immobilized Protein G (10 μL)‐Sepharose at 4 °C overnight. After washing with lysis buffer, the slurry was boiled at 95 °C for 5 min with sodium dodecyl sulfate (SDS) sample buffer. The samples were centrifuged, and supernatants were subjected to SDS/PAGE analysis followed by electroblotting analysis onto a PVDF membrane as described in western blot.

### Immunocytochemistry

Cells were seeded onto coverglasses in 6‐well culture plates. Two days later, the cells were fixed with methanol for 6 min at −20 °C. Then, cells were blocked with 5% FBS/PBS for 1 h at 37 °C. After washing with PBS, the cells were incubated with anti‐TARM1 antibodies (4 μg·mL^−1^) or anti‐DYKDDDDK mAb (1 : 100, L5, #637301; BioLegend) for 1 h at 37 °C followed by Rhodamine‐anti‐human IgG (H + L) antibody (1 : 100, #SA00007‐10; Proteintech) or AF594‐anti‐rat IgG (minimal x‐reactivity) antibody (1 : 100, Poly4054, #405422; BioLegend) for 1 h at 37 °C. Coverglasses were mounted with VECTASHIELD Antifade Mounting Medium with DAPI (#H‐1200; VECTOR LABORATORIES, Newark, NJ, USA) and observed using a ZEISS LSM980 confocal microscope. Images were acquired and processed using the zen 3 software (Zeiss, Oberkochen, Germany).

### Biolayer interferometry (BLI) analysis

Binding assays were performed using the Octet R4 system (Sartorius, Göttingen, Germany) with SA biosensors (#18‐5019; Sartorius). All steps were performed in 96‐well black microplates (#781508; Merck, Darmstadt, Germany) at 30 °C with shaking at 1000 rpm. TARM1‐Fc was biotinylated using Biotin Labeling Kit‐NH_2_ (LK03; Dojindo, Tokyo, Japan). Biotin‐TARM1‐Fc was diluted to 0.4 μg·mL^−1^ in assay buffer (PBS supplemented with 0.005% Tween20 and 0.02% BSA). Serially diluted anti‐TARM1 mAb solutions (1.56–12.5 μm) were prepared. Binding assays consisted of the following steps: First Baseline, assay buffer for 60 s; Loading, 5 μg·mL^−1^ biotin‐TARM1‐Fc for 300 s; Second Baseline, assay buffer for 60 s; Association, analyte for 900 s; and Dissociation, assay buffer for 600 s. The resulting association curve (0–900 s) and dissociation curve (0–600 s) were analyzed using the octet analysis studio 12.2.2.26 software (Sartorius). The data were fitted with a 1 : 1 binding model to obtain the *K*_D_.

### Cell stimulation

Anti‐TARM1 Abs, anti‐DYKDDDDK Ab (L5, #637301; BioLegend) or human IgG1 (all used at 5 μg·mL^−1^) were precoated on 96‐well plates. Cells (1–5 × 10^5^/well) were incubated at 37 °C for 18–24 h. Cell signaling was analyzed by flow cytometry.

## Results

### Enrichment of TARM1‐binding, scFv‐expressing yeast cells by yeast display

**Fig. 1:**
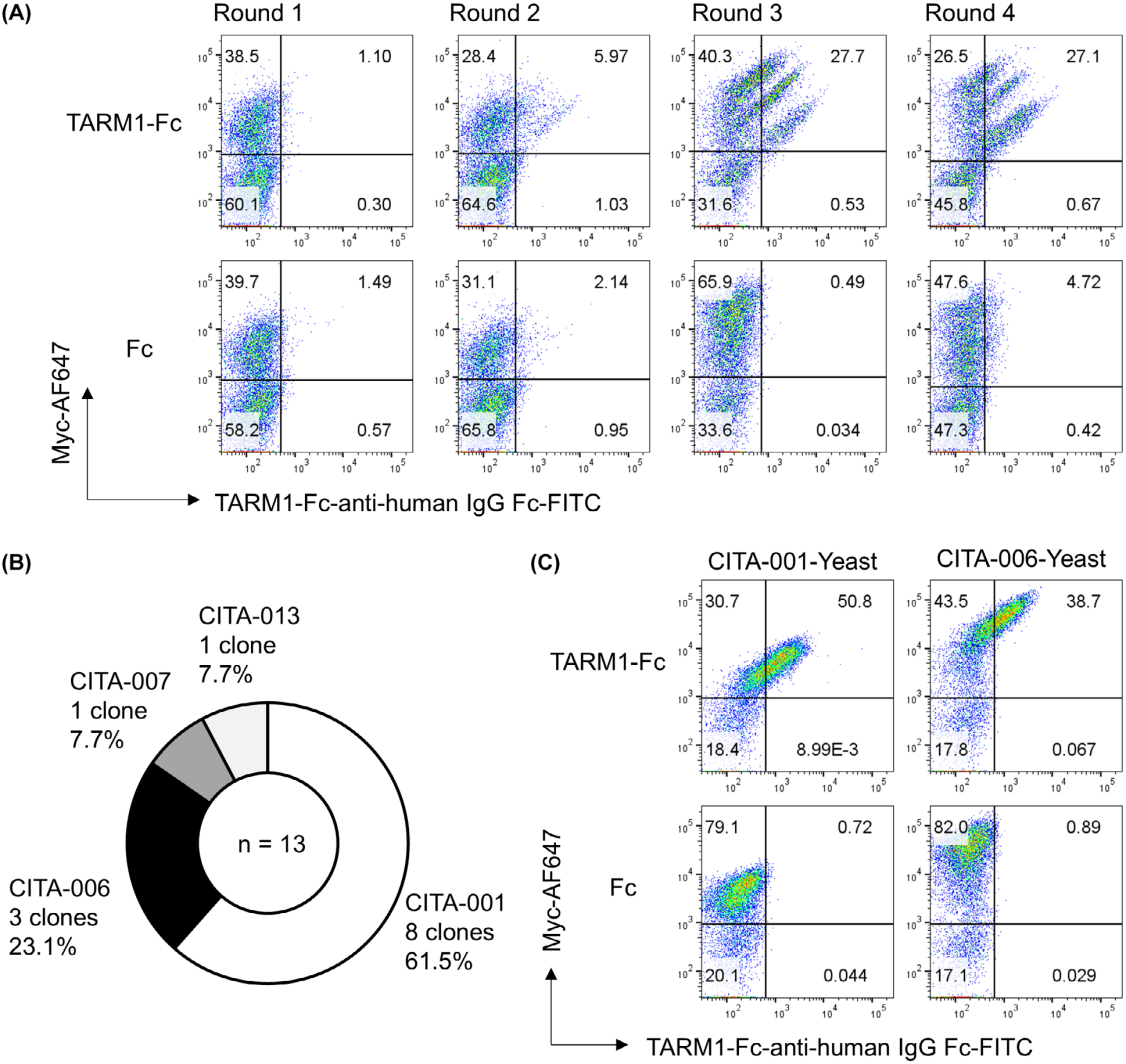
Screening of yeast cells expressing human scFvs against TARM1‐Fc. (A) The TARM1‐binding scFv yeast library was enriched by MACS (round 1, 2, and 4) or fluorescence‐activated cell sorting (FACS; round 3) as described in the Material and methods. Yeast cells in each round were incubated with TARM1‐Fc or Fc followed by FITC‐goat anti‐human IgG Fc antibody and AF647‐anti‐Myc tag antibody to detect scFv. TARM1‐Fc‐binding and Myc‐tag expression of induced yeast cells were analyzed by flow cytometry. Data are representative of two independent binding analyses performed after sorting. (B) The pie chart shows the percentage of the indicated clones among all sequenced clones. (C) Anti‐TARM1 scFvs CITA‐001‐ or CITA‐006‐expressing yeast cells were incubated with TARM1‐Fc or Fc followed by FITC‐goat anti‐human IgG Fc antibody and AF647‐anti‐Myc tag antibody to detect scFv. TARM1‐Fc‐binding and Myc expression of induced yeast cells were analyzed by flow cytometry. Data are representative of two independent experiments. CITA, Clone isolated by TARM1.

To isolate human mAbs capable of binding to TARM1, we employed a yeast display platform combined with a human scFv library. The library was subjected to four rounds of selection using recombinant human TARM1‐Fc as a target antigen: In rounds 1 and 2, magnetic‐activated cell sorting (MACS) was performed to enrich scFv‐expressing yeast cells capable of binding TARM1‐Fc. In round 3, FACS was employed to highly select specific TARM1‐binding yeast cells. In round 4, an additional MACS step was performed to enrich the yeast binders. After each selection, the yeast population was analyzed by flow cytometry using TARM1‐Fc labeled with FITC‐conjugated anti‐human IgG Fc antibody and AF647‐conjugated anti‐Myc antibody. As shown in Fig. 1A, the population of yeast clones expressing scFv and binding to TARM1‐Fc (scFv^+^TARM1‐Fc^+^) increased after each selection round.

After the fourth round of selection, plasmids were extracted from 13 randomly selected yeast clones. They were sequenced to determine their amino acid sequences. The isolated clones were designated as CITA (Clone Isolated by TARM1) followed by clone numbers (e.g., CITA‐001). Sequence alignment of scFvs showed that eight clones were identical (CITA‐001; 61.5%). Three clones (CITA‐006; 23.1%) shared a second unique sequence. The remaining two clones each (CITA‐007 and CITA‐013; 7.7%) carried distinct sequences (Fig. 1B). Notably, all 13 clones possessed lambda light chain variable regions. To examine antigen binding, scFv CITA‐001 or CITA‐006‐expressing yeast clones were analyzed by flow cytometry. Both yeast clones specifically bound to TARM1‐Fc, but not to Fc (Fig. 1C).

### Anti‐TARM1 mAbs CITA‐001 and CITA‐006 bind to TARM1‐fc

**Fig. 2:**
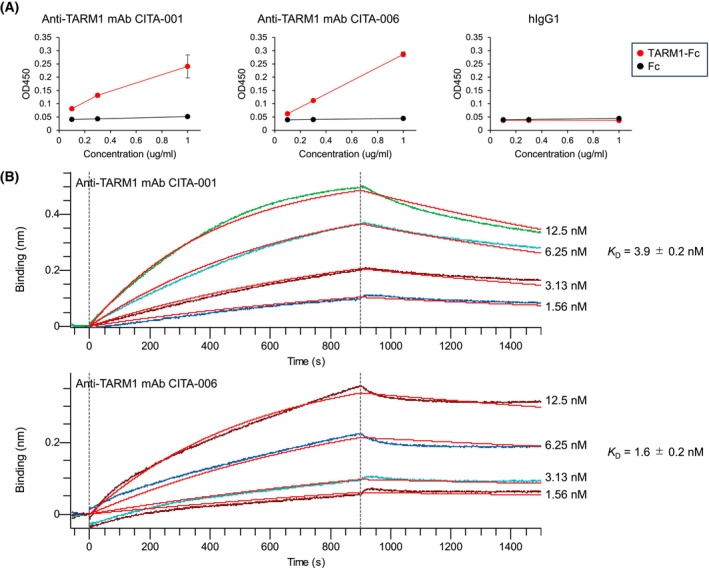
Anti‐TARM1 mAbs CITA‐001 and CITA‐006 bind to TARM1‐Fc. (A) Biotin‐TARM1‐Fc (Red) or Biotin‐Fc (Black) on anti‐TARM1 mAbs CITA‐001 or CITA‐006‐coated plates were incubated with Streptavidin‐HRP and TMB substrate. hIgG1 was used as a control. Absorbance was measured using a microplate reader. Data are representative of two independent experiments. Mean ± SD of triplicate wells. (B) Representative graph of BLI sensorgrams of anti‐TARM1 mAbs CITA‐001 (upper) and CITA‐006 (lower) against biotin‐TARM1‐Fc immobilized on streptavidin‐sensors. Sensorgrams of bold lines indicate different concentrations of anti‐TARM1 mAbs CITA‐001 and CITA‐006, and the fitting curves are shown as red lines. Data are representative of three independent experiments. CITA, Clone isolated by TARM1.

To generate full‐length mAbs, the VH and VL chain sequences of scFvs CITA‐001 and CITA‐006 were cloned into mammalian expression vectors encoding the constant regions of human IgHG1 and lambda light chain, respectively. The resulting plasmids were co‐transfected into Expi293F cells for transient antibody expression. mAbs were obtained from culture supernatants by affinity chromatography. The purified antibodies were evaluated for binding to TARM1‐Fc by sandwich ELISA. Both anti‐TARM1 mAbs CITA‐001 and CITA‐006 bound to TARM1‐Fc in a dose‐dependent manner (Fig. 2A).

To determine binding affinity of anti‐TARM1 mAbs CITA‐001 and CITA‐006, we performed a BLI‐based binding assay. Biotinylated TARM1‐Fc was immobilized onto Streptavidin‐biosensors, and binding kinetics were assessed by exposing the sensors to serially diluted anti‐TARM1 mAbs CITA‐001 and CITA‐006. The association and dissociation curves were fitted to a 1 : 1 binding model, and the equilibrium dissociation constants (*K*_D_) were calculated. The *K*_D_ value for anti‐TARM1 mAb CITA‐001 was 3.9 ± 0.2 nm, while that for CITA‐006 was 1.6 ± 0.2 nm, respectively (Fig. 2B).

### Anti‐TARM1 mAbs CITA‐001 and CITA‐006 bind to cell‐surface TARM1

**Fig. 3:**
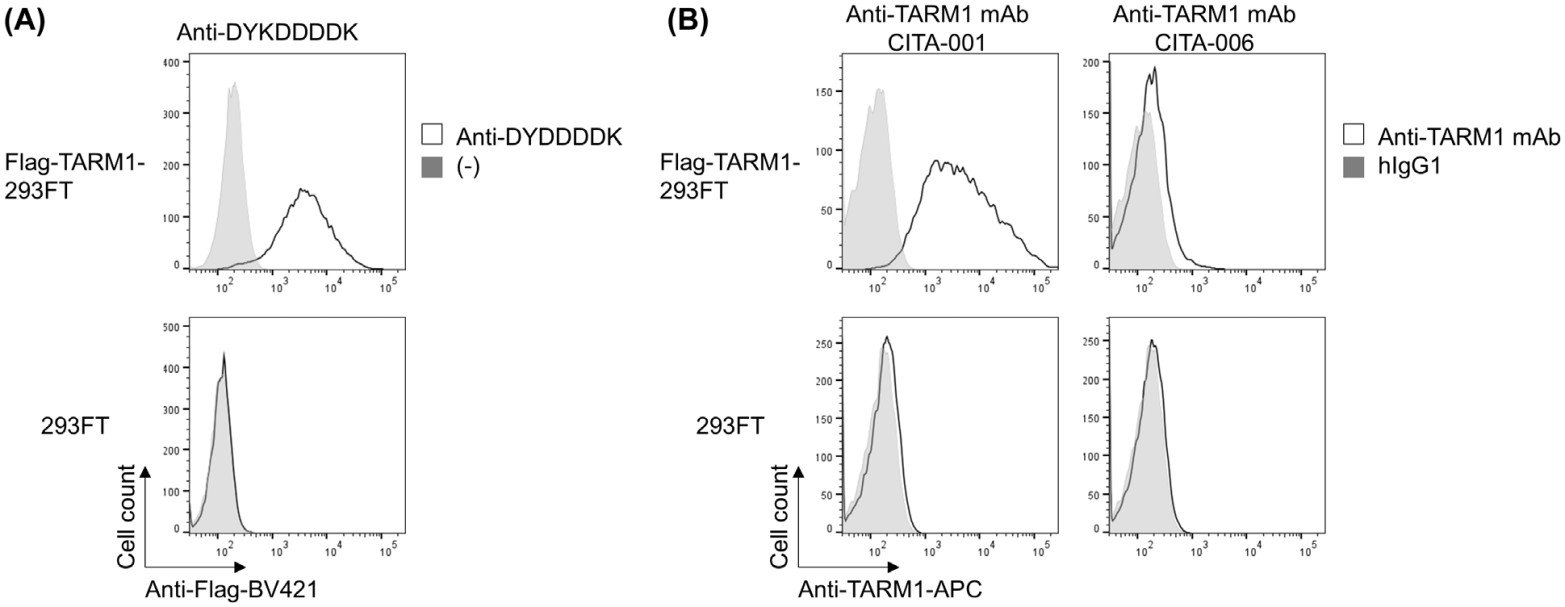
Anti‐TARM1 mAbs CITA‐001 and CITA‐006 bind to cell‐surface TARM1. (A) Expression of Flag‐TARM1 on 293FT cells was analyzed by flow cytometry with BV421‐anti‐Flag antibody. Data are representative of three independent experiments. (B) Binding of anti‐TARM1 mAbs CITA‐001 and CITA‐006 to Flag‐TARM1‐293FT or 293FT cells was analyzed by flow cytometry. hIgG1 was used as a control. Data are representative of three independent experiments. CITA, Clone isolated by TARM1.

To determine whether anti‐TARM1 mAbs CITA‐001 and CITA‐006 recognize cell‐surface TARM1, we first generated 293FT cells stably‐expressing Flag‐tagged TARM1 by a retroviral vector system. Cell‐surface expression of Flag‐TARM1 was confirmed by flow cytometry with anti‐DYKDDDDK antibody (Fig. 3A). We then analyzed binding of anti‐TARM1 mAbs CITA‐001 and CITA‐006 to Flag‐TARM1‐293FT and 293FT cells by flow cytometry. Anti‐TARM1 mAbs CITA‐001 and CITA‐006 differed in their ability to recognize cell‐surface TARM1 (Fig. 3B). CITA‐001 showed an apparent shift in APC fluorescence for Flag‐TARM1‐293FT cells. In contrast, CITA‐006 exhibited only a modest increase in APC signal to Flag‐TARM1‐293FT cells, suggesting its relatively weak affinity for cell‐surface TARM1. Nonetheless, this weak shift was reproducibly observed across multiple independent experiments and consistently high compared with an isotype control.

### Anti‐TARM1 mAbs CITA‐001 and CITA‐006 are not suitable for TARM1 in western blotting, immunoprecipitation or immunocytochemistry

We assessed the applicability of anti‐TARM1 mAbs CITA‐001 and CITA‐006 as probing reagents in biochemical studies: Western blot, immunoprecipitation and immunocytochemistry. First, we examined whether anti‐TARM1 mAbs CITA‐001 and CITA‐006 could detect TARM1 by western blotting. WCLs of Flag‐TARM1‐expressing 293FT cells and parental 293FT cells were analyzed by SDS/PAGE followed by immunoblotting with anti‐TARM1 mAbs and an anti‐DYKDDDDK monoclonal antibody. Anti‐TARM1 mAbs CITA‐001 and CITA‐006 showed no detectable band corresponding to Flag‐TARM1 (approximately 40 kDa) under the experimental conditions (Fig. S1A). In contrast, the anti‐DYKDDDDK antibody detected a band at the expected molecular weight of TARM1, confirming that Flag‐TARM1 protein was detectable under the assay conditions. Anti‐DYKDDDDK antibody also detected an additional band at approximately 65 kDa in Flag‐TARM1‐expressing cells, but not in parental 293FT cells. It is likely that the additional band corresponds to the uncleaved fusion form of Flag‐TARM1‐T2A‐emGFP.

Second, we examined whether the anti‐TARM1 mAbs CITA‐001 or CITA‐006 could immunoprecipitate Flag‐TARM1 protein. WCLs of Flag‐TARM1‐293FT cells were incubated with antibody‐conjugated Protein G‐beads. The resulting precipitates were analyzed by immunoblotting with anti‐DYKDDDDK antibody. Anti‐TARM1 mAbs CITA‐001 and CITA‐006 did not recover TARM1 protein (Fig. S1B). In contrast, the anti‐DYKDDDDK antibody efficiently precipitated Flag‐TARM1 under the same conditions.

Third, we examined whether the anti‐TARM1 mAbs CITA‐001 and CITA‐006 could be used as probes for immunocytochemistry. TARM1‐expressing 293FT and 293FT cells were fixed and stained with anti‐TARM1 mAbs or anti‐DYKDDDDK antibody followed by Rhodamine‐anti‐human IgG antibody or AF594‐anti‐rat IgG antibody. The signal was observed with a confocal microscope. Anti‐TARM1 mAbs CITA‐001 and CITA‐006 did not show any specific staining signals when compared with the 293FT cell background (Fig. S1C). In contrast, anti‐DYKDDDDK antibody exhibited apparent staining of TARM1‐expressing HEK293FT cells, but not of parental 293FT cells, confirming that the Flag‐tagged TARM1 protein was detectable by immunocytochemistry under the assay conditions.

### Anti‐TARM1 mAbs CITA‐001 and CITA‐006 induce activation signals into reporter cells

**Fig. 4:**
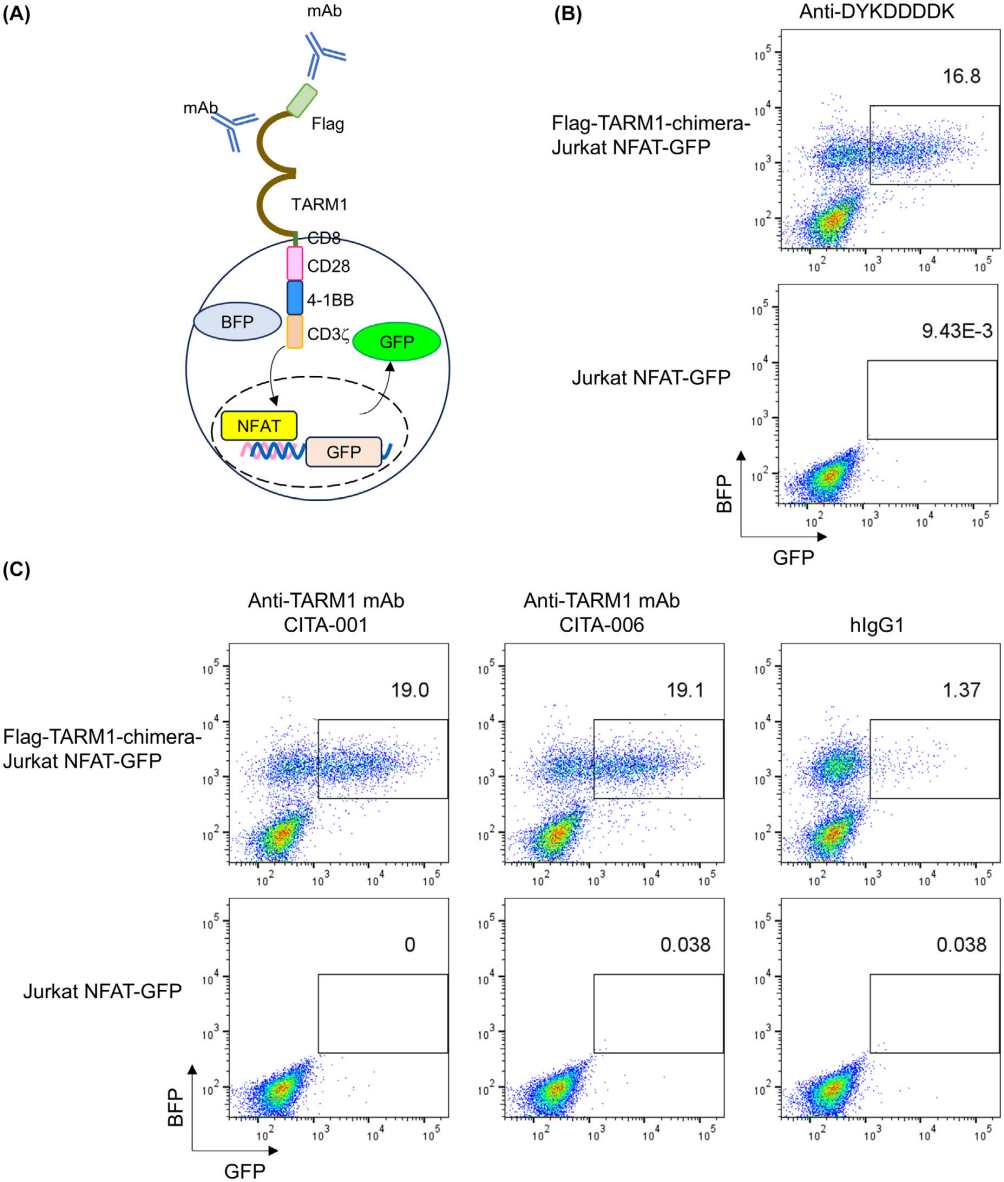
Anti‐TARM1 mAbs CITA‐001 and CITA‐006 induce signals into reporter cells. (A) Schematic illustration of Flag‐TARM1‐chimera‐Jurkat NFAT‐GFP cells. (B) Flag‐TARM1‐chimera‐Jurkat NFAT‐GFP or parental Jurkat NFAT‐GFP cells were incubated with plate‐coated anti‐DYKDDDDK antibody for 18 h. GFP expression was analyzed by flow cytometry. BFP^+^ cells represent TARM1‐chimera–expressing cells via the T2A sequence and were gated for analysis. Data are representative of three independent experiments. (C) Flag‐TARM1‐chimera‐Jurkat NFAT‐GFP and parental Jurkat NFAT‐GFP cells were incubated with plate‐coated anti‐TARM1 mAbs CITA‐001, CITA‐006, or hIgG1 as an isotype control for 18 h. GFP expression was analyzed by flow cytometry. BFP^+^ cells represent TARM1‐chimera–expressing cells via the T2A sequence and were gated for analysis. Data are representative of three independent experiments. CITA, Clone isolated by TARM1. BFP, monomeric blue fluorescent protein with improved brightness and chemical stability; GFP, Green fluorescent protein; NFAT, Nuclear factor of activated T cells.

To evaluate whether these antibodies possessed agonistic activity, we constructed Jurkat NFAT‐GFP reporter cells stably expressing a Flag‐tagged TARM1 fused with CD28–4‐1BB–CD3ζ signaling domains and T2A‐BFP (Flag‐TARM1‐chimera–Jurkat NFAT‐GFP cells) using a retroviral vector system (Figs 4A and S2). In the assay, stimulation of the chimeric receptor activates NFAT‐dependent transcription, resulting in GFP expression that serves as an indicator of agonistic signaling. Cross‐linking of the chimeric receptor with anti‐DYKDDDDK antibody induced GFP expression, confirming that the chimeric receptor configuration was capable of transducing activation signals (Fig. 4B). When these cells were stimulated with anti‐TARM1 mAbs, GFP expression was induced in BFP^+^ Flag‐TARM1‐chimera–Jurkat NFAT‐GFP cells, but not in parental Jurkat NFAT‐GFP cells. An isotype‐matched IgG1 control antibody failed to elicit GFP induction, indicating the specificity of the agonistic response (Fig. 4C).

We further examined whether the anti‐TARM1 mAbs CITA‐001 and CITA‐006 could promote activation of primary human moDCs. Human moDCs were stimulated with plate‐coated antibodies, and the surface expression of HLA‐DR and CD86 activation markers was assessed by flow cytometry. Under these experimental conditions, anti‐TARM1 mAb CITA‐006 induced a modest upregulation of HLA‐DR and CD86 expression compared with PBS‐treated moDCs, whereas anti‐TARM1 mAb CITA‐001 didn't. Notably, a comparable upregulation of HLA‐DR and CD86 was also detected in moDCs treated with human IgG1 control. Consequently, we could not determine whether our antibodies possessed agonist‐like activity in moDCs.

## Discussion

In this study, we generated novel fully human mAbs against TARM1 by a yeast display library expressing human scFvs. Through iterative rounds of selection, we obtained two clones, CITA‐001 and CITA‐006, that selectively bound to human TARM1‐Fc and recognized TARM1 expressed on the surface of 293FT cells. Also, these antibodies could trigger activation signaling into Jurkat NFAT‐GFP reporter cells via TARM1 engagement. These results indicate that these antibodies not only serve as valuable tools for dissecting TARM1 biology but also act as potential agents for activating TARM1‐mediated immune responses.

Therapeutic strategies targeting LILRs have been explored in various immune‐mediated contexts [1, 5, 6]. However, many of these antibodies were initially generated as murine mAbs, requiring subsequent humanization steps before clinical application, which can limit their direct translational utility. In the present study, we generated fully human mAbs against TARM1 by combining a fully human scFv library with a yeast display platform. This approach enables efficient isolation of human antibodies, facilitating their potential translation into clinical applications.

Importantly, activating immune receptors have emerged as promising immuno‐oncology targets [19], particularly for patients who do not respond to immune checkpoint inhibitors. Agonistic antibodies against receptors such as CD40 have advanced to clinical evaluation [20], suggesting the potential to boost antitumor immunity by promoting antigen presentation and T cell activation. In this context, TARM1, as such an activating myeloid receptor, may contribute to reprogram tumor‐associated myeloid cells toward a proinflammatory, antitumor phenotype. Given the increasing recognition of the role of myeloid cells in shaping the tumor microenvironment, TARM1 agonists could complement checkpoint blockade strategies, potentially expanding the proportion of patients who benefit from immunotherapy.

In functional analyses, we showed the utility of our anti‐TARM1 mAbs in conventional immunological assays. These antibodies were not applicable for western blotting, immunoprecipitation, or immunocytochemistry, likely due to their inability to recognize denatured or fixed TARM1 and their preferential binding to native conformations. Although we attempted to evaluate their agonistic activity using moDCs, control human IgG1 itself elicited agonist‐like effects. This observation suggests that the activation was likely to be mediated through Fc receptor (FcR) engagement rather than specific recognition of TARM1. These results suggest that while our mAbs provide valuable tools for probing TARM1 function in engineered reporter systems, their application in primary myeloid cell assays or standard protein detection platforms requires further optimization, such as Fc engineering or assay redesign.

Although anti‐TARM1 mAbs CITA‐001 and CITA‐006 showed binding to human TARM1, species cross‐reactivity remains to be determined. Sequence alignment indicates that the extracellular domains of human and murine TARM1 share only moderate amino acid identity (approximately 47%), suggesting that our mAbs are likely specific for human TARM1. Evaluating cross‐reactivity will be important for future *in vivo* studies, especially those involving mouse models of immune regulation or cancer immunotherapy.

In conclusion, we developed fully human mAbs against TARM1, with high specificity and agonistic activity. These antibodies represent not only important tools for basic research but also potential candidates for developing novel therapeutics in the treatment of immune‐mediated diseases and cancer.

## Conflict of interest

RY and HT declare that patent applications related to the anti‐TARM1 antibodies described in this study are under consideration. The authors declare no other conflict interest.

## Author contributions

RY and HT conceived and supervised the study. RY designed experiments; RY, MS, MH, KA and SO performed experiments and analyzed data; RY wrote the manuscript; MS, MH and HT made manuscript revisions.

## Supporting information


**Fig. S1.** Anti‐TARM1 mAbs CITA‐001 and CITA‐006 are not suitable for TARM1 in western blotting, immunoprecipitation or immunocytochemistry.
**Fig. S2.** Amino acid sequence of Flag‐tagged TARM1 fused with CD28–4‐1BB–CD3ζ signaling.

## Data Availability

The data that support the findings of this study are available from the corresponding author (tanno-hd@igakuken.or.jp) upon reasonable request. The amino acid sequences of the anti‐TARM1 monoclonal antibodies CITA‐001 and CITA‐006 have been deposited in the AntiBodies Chemically Defined (ABCD) database, with the accession numbers ABCD_BG485 for CITA‐001 and ABCD_BG486 for CITA‐006. Due to ongoing patent considerations, the sequences are designated as non‐disclosable in ABCD and will be kept confidential during the patent filing process. Anti‐TARM1 monoclonal antibodies described in this study are available from the corresponding author upon reasonable request and subject to a material transfer agreement (MTA), for the purpose of independent validation during the patent filing period.
