# Supplementary material for: Development of human monoclonal antibodies against TARM1 by yeast display
Source: FEBS Open Bio. 2026 Apr 9;16(7):1358–69. doi: 10.1002/2211-5463.70216 (PMC13327005; doi:10.1002/2211-5463.70216)
Supplement: Supplementary file 1 — Fig. S1. Anti‐TARM1 mAbs CITA‐001 and CITA‐006 are not suitable for TARM1 in western blotting, immunoprecipitation or immunocytochemistry. Fig. S2. Amino acid sequence of Flag‐tagged TARM1 fused with CD28–4‐1BB–CD3ζ signaling. [file FEB4-16-1358-s001.pptx]

## Slide 1
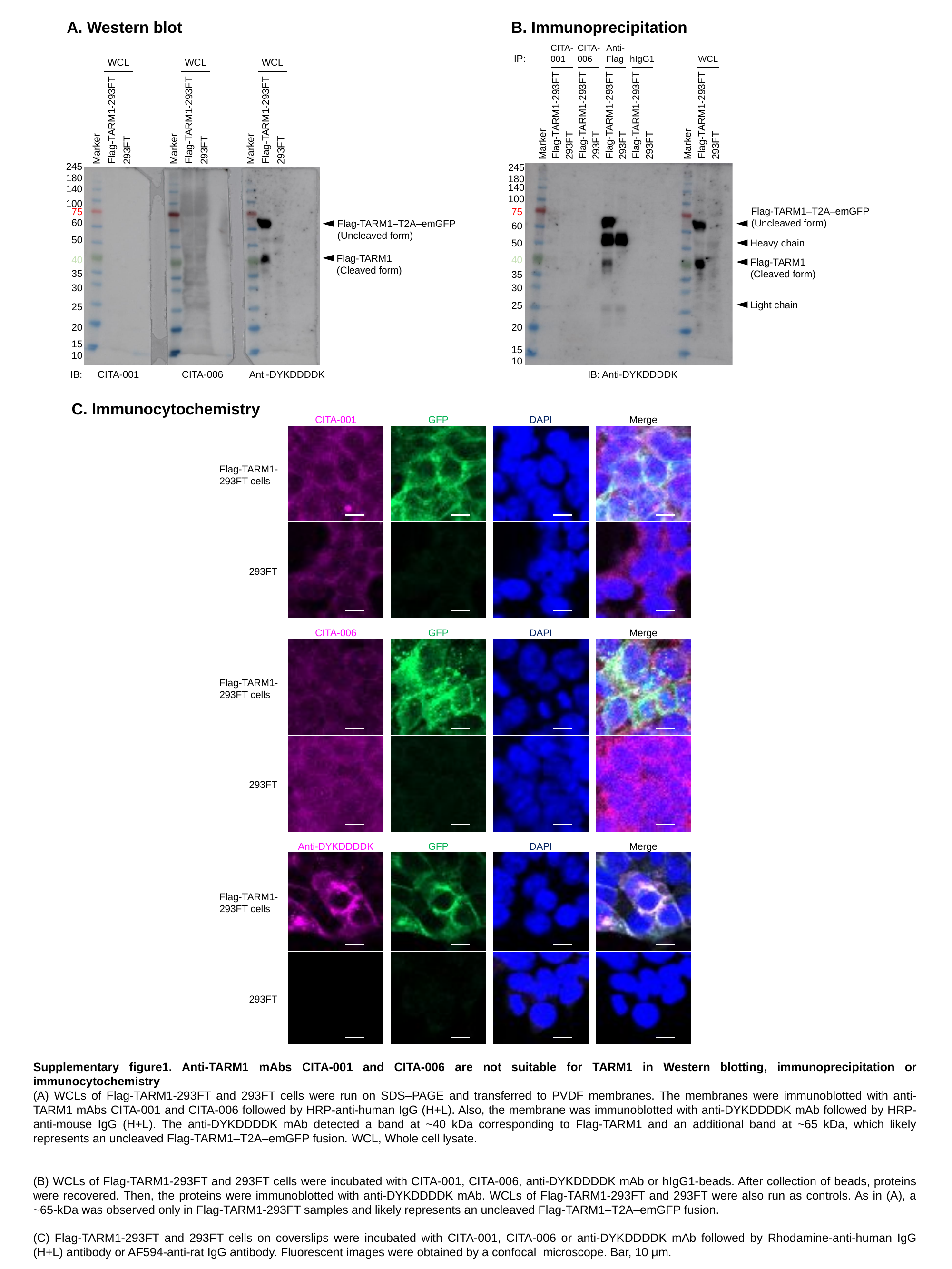

A. Western blot
B. Immunoprecipitation
CITA-
001
CITA-
006
Anti-
Flag
IP:
hIgG1
WCL
WCL
Flag-TARM1-293FT
Marker
293FT
WCL
Flag-TARM1-293FT
Marker
293FT
WCL
Flag-TARM1-293FT
Marker
293FT
Flag-TARM1-293FT
Flag-TARM1-293FT
Flag-TARM1-293FT
Flag-TARM1-293FT
Flag-TARM1-293FT
Marker
Marker
293FT
293FT
293FT
293FT
293FT
245
245
180
180
140
140
100
100
Flag-TARM1–T2A–emGFP
(Uncleaved form)
75
75
60
Flag-TARM1–T2A–emGFP
(Uncleaved form)
60
50
50
Heavy chain
Flag-TARM1
(Cleaved form)
40
40
Flag-TARM1
(Cleaved form)
35
35
30
30
Light chain
25
25
20
20
15
15
10
10
IB:
CITA-001
CITA-006
Anti-DYKDDDDK
IB: Anti-DYKDDDDK
C. Immunocytochemistry
CITA-001
GFP
DAPI
Merge
Flag-TARM1-
293FT cells
293FT
CITA-006
GFP
DAPI
Merge
Flag-TARM1-
293FT cells
293FT
Anti-DYKDDDDK
GFP
DAPI
Merge
Flag-TARM1-
293FT cells
293FT
Supplementary figure1. Anti-TARM1 mAbs CITA-001 and CITA-006 are not suitable for TARM1 in Western blotting, immunoprecipitation or immunocytochemistry
(A) WCLs of Flag-TARM1-293FT and 293FT cells were run on SDS–PAGE and transferred to PVDF membranes. The membranes were immunoblotted with anti-TARM1 mAbs CITA-001 and CITA-006 followed by HRP-anti-human IgG (H+L). Also, the membrane was immunoblotted with anti-DYKDDDDK mAb followed by HRP-anti-mouse IgG (H+L). The anti-DYKDDDDK mAb detected a band at ~40 kDa corresponding to Flag-TARM1 and an additional band at ~65 kDa, which likely represents an uncleaved Flag-TARM1–T2A–emGFP fusion. WCL, Whole cell lysate.
(B) WCLs of Flag-TARM1-293FT and 293FT cells were incubated with CITA-001, CITA-006, anti-DYKDDDDK mAb or hIgG1-beads. After collection of beads, proteins were recovered. Then, the proteins were immunoblotted with anti-DYKDDDDK mAb. WCLs of Flag-TARM1-293FT and 293FT were also run as controls. As in (A), a ~65-kDa was observed only in Flag-TARM1-293FT samples and likely represents an uncleaved Flag-TARM1–T2A–emGFP fusion.
(C) Flag-TARM1-293FT and 293FT cells on coverslips were incubated with CITA-001, CITA-006 or anti-DYKDDDDK mAb followed by Rhodamine-anti-human IgG (H+L) antibody or AF594-anti-rat IgG antibody. Fluorescent images were obtained by a confocal microscope. Bar, 10 μm.

## Slide 2
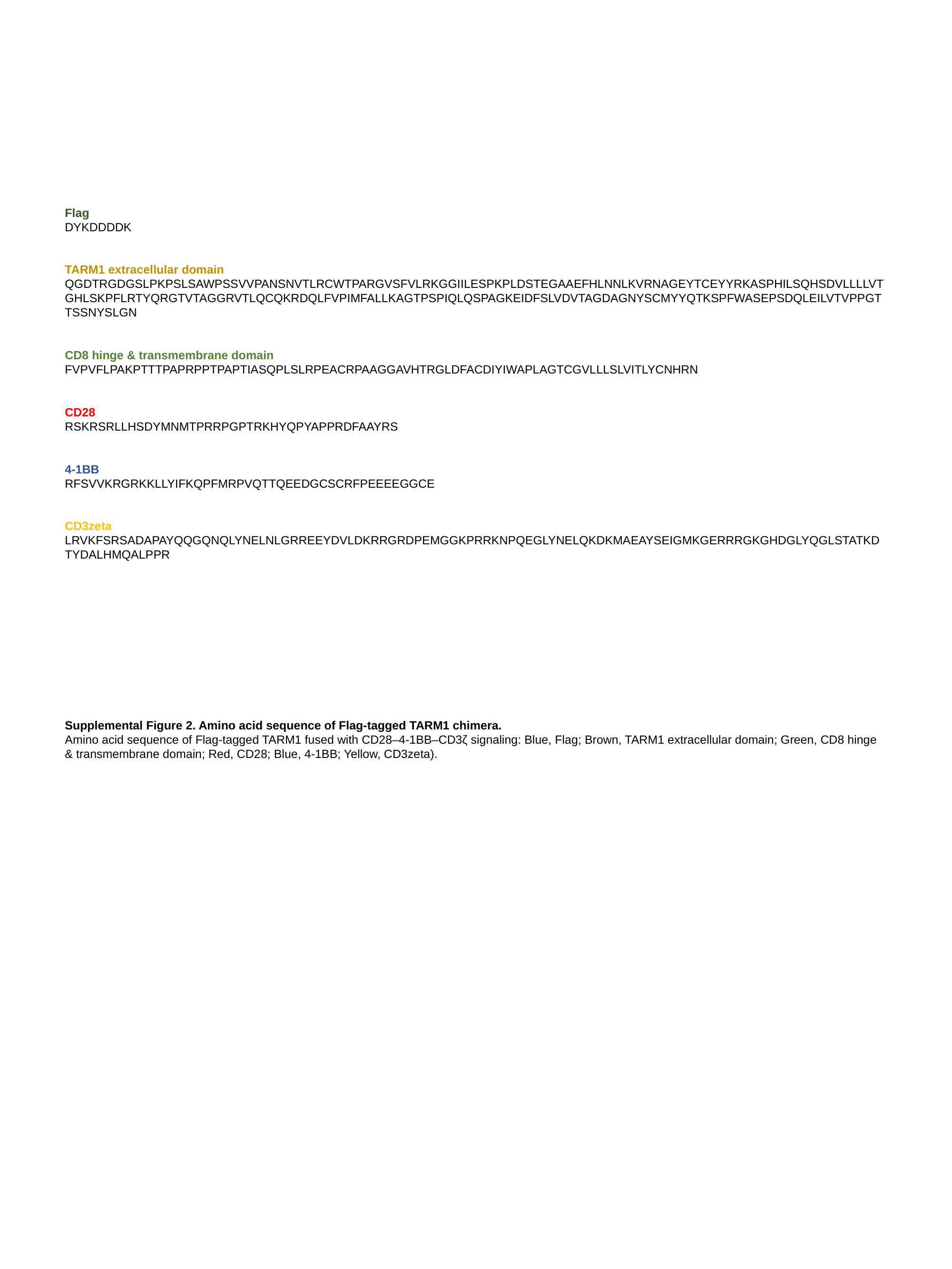

Flag
DYKDDDDK
TARM1 extracellular domain
QGDTRGDGSLPKPSLSAWPSSVVPANSNVTLRCWTPARGVSFVLRKGGIILESPKPLDSTEGAAEFHLNNLKVRNAGEYTCEYYRKASPHILSQHSDVLLLLVTGHLSKPFLRTYQRGTVTAGGRVTLQCQKRDQLFVPIMFALLKAGTPSPIQLQSPAGKEIDFSLVDVTAGDAGNYSCMYYQTKSPFWASEPSDQLEILVTVPPGTTSSNYSLGN
CD8 hinge & transmembrane domain
FVPVFLPAKPTTTPAPRPPTPAPTIASQPLSLRPEACRPAAGGAVHTRGLDFACDIYIWAPLAGTCGVLLLSLVITLYCNHRN
CD28
RSKRSRLLHSDYMNMTPRRPGPTRKHYQPYAPPRDFAAYRS
4-1BB
RFSVVKRGRKKLLYIFKQPFMRPVQTTQEEDGCSCRFPEEEEGGCE
CD3zeta
LRVKFSRSADAPAYQQGQNQLYNELNLGRREEYDVLDKRRGRDPEMGGKPRRKNPQEGLYNELQKDKMAEAYSEIGMKGERRRGKGHDGLYQGLSTATKDTYDALHMQALPPR
Supplemental Figure 2. Amino acid sequence of Flag-tagged TARM1 chimera.
Amino acid sequence of Flag-tagged TARM1 fused with CD28–4-1BB–CD3ζ signaling: Blue, Flag; Brown, TARM1 extracellular domain; Green, CD8 hinge & transmembrane domain; Red, CD28; Blue, 4-1BB; Yellow, CD3zeta).
